# Supplementary material for: Enhanced oxidative stress and damage in glycated erythrocytes
Source: PLoS One. 2020 Jul 6;15(7):e0235335. doi: 10.1371/journal.pone.0235335 (PMC7337333; doi:10.1371/journal.pone.0235335)
Supplement: S1 File — (DOCX) [file pone.0235335.s001.docx]

**Supporting Information**

original, uncropped and unadjusted images underlying all blot results
