# Supplementary material for: Enhanced oxidative stress and damage in glycated erythrocytes
Source: PLoS One. 2020 Jul 6;15(7):e0235335. doi: 10.1371/journal.pone.0235335 (PMC7337333; doi:10.1371/journal.pone.0235335)
Supplement: S1 Fig — Representative figures of the mass spectra obtained in three independent experiments for each incubation condition: 0 (G0), 5 (G5), 25 (G25) and 137 (G137) mmol/l glucose. On each spectrum, four main peaks were obtained corresponding to α-hemoglobin (αHb; 15130 Da), glycated α-hemoglobin (gαHb; 15330 Da), β-hemoglobin (βHb; 15890 Da), glycated β-hemoglobin (gβHb; 16100 Da). (DOCX) [file pone.0235335.s002.docx]

**Fig S1**

**
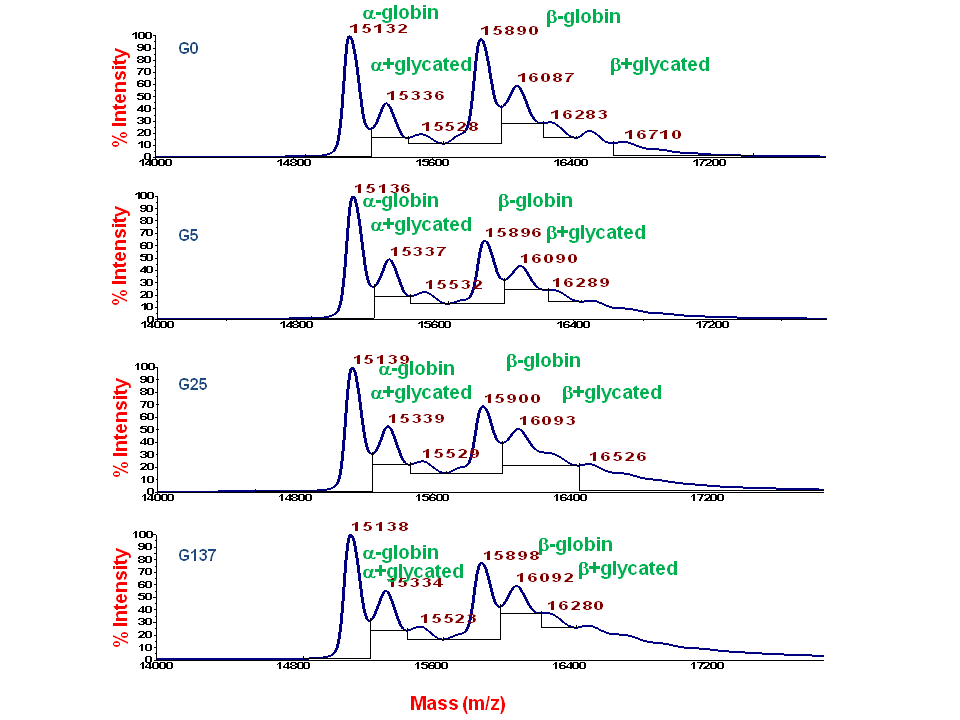
**

**Figure S1: Characterisation of glycation percentage in the different erythrocyte preparations by mass spectrometry.**

Representative figures of the mass spectra obtained in three independent experiments for each incubation condition: 0 (G0), 5 (G5), 25 (G25) and 137 (G137) mmol/l glucose. On each spectrum, four main peaks were obtained corresponding to α-hemoglobin (αHb; 15130 Da), glycated α-hemoglobin (gαHb; 15330 Da), β-hemoglobin (βHb; 15890 Da), glycated β-hemoglobin (gβHb; 16100 Da).
