# Supplementary material for: Enhanced oxidative stress and damage in glycated erythrocytes
Source: PLoS One. 2020 Jul 6;15(7):e0235335. doi: 10.1371/journal.pone.0235335 (PMC7337333; doi:10.1371/journal.pone.0235335)
Supplement: S2 Fig — Typical Forward Scatter (FSC) and Side Scatter (SSC) characteristics represented in dot-blot graph obtained by cytometry erythrocytes from non diabetic (left) and diabetic persons (right). (DOCX) [file pone.0235335.s003.docx]

**Fig S2**


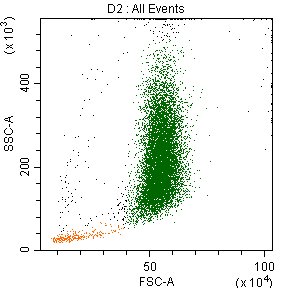

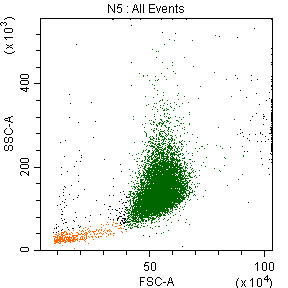


**ND**

**D**

**Figure S2. Diabetes alters erythrocyte morphology.**

Typical Forward Scatter (FSC) and Side Scatter (SSC) characteristics represented in dot-blot graph obtained by cytometry erythrocytes from non diabetic (left) and diabetic persons (right).
