# Supplementary material for: Enhanced oxidative stress and damage in glycated erythrocytes
Source: PLoS One. 2020 Jul 6;15(7):e0235335. doi: 10.1371/journal.pone.0235335 (PMC7337333; doi:10.1371/journal.pone.0235335)
Supplement: Attachment — Submitted filename: Response to rev June 5th 2020.docx [file pone.0235335.s004.docx]

**Reviewer #2:**

Majors:

In the hemolysis study, the results indicate that low concentrated glucose incubation had a protective membrane-stabilizing effect, which means an increased “flexibility” of the cell membrane or less fragile membrane. However, in the test of deformability, elongation index was reduced after glucose incubation, which suggests a reduced “flexibility” of the cell membrane and/or cytoskeleton. How does the authors explain these possible “conflict” phonotypes?

It is true, erythrocytes incubated with low glucose concentration exhibit an enhanced resistance to free radical-induced hemolysis concomitantly with an impaired flexibility of their membrane. Then, results obtained in the hemolysis and deformability tests may appear conflictual. Actually, these two tests did not assess the same biological phenomenon. Our *in vitro* free radical-induced blood hemolysis test allows determination of erythrocyte capacity to resist to an oxidative stress mediated by a free radical generator (AAPH), a chemical agent. Results were expressed as the half time of hemolysis (HT50) and reported on figure 2E.

The deformability of the different erythrocyte preparations was analysed by ektacytometry. This methodology allows elongation index determination, a biophysical index which expresses erythrocyte deformability capacity in response to increasing shear stress (Fig 2F and 2G).

Hence, if our “hemolysis test” measures erythrocyte capacity to resist to hemolysis when submitted to a free radical generator, elongation index specifically expresses membrane flexibility/rigidity. In other words, these two different methods give two types of results.

Concerning results, erythrocytes that have been incubated with high glucose concentrations exhibit both impaired hemolysis resistance capacity (2E) and reduced membrane flexibility (Fig 2F and 2G).

In some papers (for example PMID: 31704555), erythrocytes from diabetes showed impaired hemolysis resistance, which is opposite the phenomenon observed in this manuscript. Do the in vitro and in vivo glycations lead to different hemolysis results?

Thank you for mentioning this.

We agree, in some papers, erythrocytes from diabetes showed impaired hemolysis resistance. Please note, the mentioned reference (PMID: 31704555) is a recent article from our group. In this article, erythrocytes from diabetic patients exhibited a slight **non-significant** decrease in free radical-induced hemolysis half time in comparison with erythrocytes from non-diabetic (Cf fig A).

**Figure 1** is issued from Catan et al (2019) *Atherosclerosis*.

Considering phenomenon observed in our present manuscript, if erythrocytes *in vitro* incubated with 5 or 25 mM of glucose (G5, G25) exhibited a higher HT50 compared to G0 (Figure 2E), those incubated with 137 mM of glucose (G137) do not exhibit such hemolysis resistance any more. In addition, HbA1c measurement in our *in vitro* glycated preparations revealed that values obtained for G137 erythrocytes (7%) were highly similar to HbA1c values observed in diabetic patients (table 1).

Hence, in our experimental conditions, *in vitro* and *in vivo* glycation lead to similar hemolysis results.

Finally, please note in our present study, a slight non-significant impairment in HT50 for erythrocytes was also observed when isolated from diabetics when compared to non-diabetic (Cf figure 2).

**Figure 2.** This figure was established with data from the present study.

The high glucose concentration may change the osmolarity of the solution and making the solution hypertonic, therefore the cells may already in a shrank state in the initial stage, which may cause influence on the results. Did the authors detect the cell morphology with microscopy?

It is true, high glucose concentration may change the osmolarity of the solution making cell potentially in a shrank state in the initial stage.

In our experimental conditions, we did not observe strong evidence of such phenomenon by microscopy (fig 3). If an increased number erythrocyte ghosts is observed when incubated with increasing glucose concentrations (see arrows), most erythrocytes does not exhibit a significant shrank state in relation to glucose concentration.

In addition, if such phenomenon would occur, please note our erythrocyte preparations used in the present MS, were washed in an isotonic solution prior analysis.

**Figure 3.** This figure corresponds to erythrocyte preparations that were incubated with 5 (A), 50 (B) or 100 (C) mM glucose for five days. Please note these preparations were not washed in an isotonic solution prior microscopic analysis. Bar corresponds to 10 µm.

The proteasome activity detected in in vitro and in vivo showed opposite results in the manuscript. When treated with high glucose level, impaired proteasome activity was detected. But the proteasome activity from the diabetic erythrocytes showed an increased activity. How does the author explain these results?

We agree with this and explanations are provided in our revised version of our MS.

Page 14 we wrote:

“Conversely, the chymotrypsin-like activity of the proteasome appeared to be significantly enhanced in erythrocytes purified from diabetics compared to those from non-diabetics (table 4). Proteasome activity may be activated under moderate oxidative stress [1]. *In vivo*, if oxidative stress is higher in erythrocytes from diabetics than from non-diabetics, no variation was measured in term of 4-HNE accumulation. Enhanced oxidative stress in *in vivo* glycated erythrocytes may be significant and moderate enough to trigger proteasome LLVY activation.”

Minors:

The data analysis used in Table2 & 3 should be One-way ANOVA.

We followed Reviewer’s advice and used One-way ANOVA for statistical analysis in table 2 and 3.

Modifications in the revised version of our MS were reported lines 236, 287 and 289.

If possible, can the author add the AOPP data back?

We followed Reviewer’s advice, page 14 we wrote “In addition, accumulation of advanced oxidation product (AOPP), was found to be higher (+18%) in erythrocytes from diabetics but this increase did not reach significance.”

In the method section, water was used to induce lysis of the erythrocytes sticking on the surface of ECs. EC adhesion is not very strong. Does this method also wash away some EC and affect the quantification of internalized erythrocytes?

Yes, we did use water to induce lysis of erythrocytes sticking on the surface of cultured endothelial cells. This method may indeed wash away some endothelial cells and such phenomenon was noticed when cells were treated with highly glycated erythrocytes. Still, this phenomenon could not affect our results since enhanced internalization of erythrocytes was significant and observed in endothelial cells that have been incubated with glycated erythrocytes. In addition, please note erythrocyte phagocytosis by endothelial cells was confirmed by recent unpublished data using flow cytometry and confocal microscopy. These results would be part of a future MS.

The figures need to be reorganized. The supplementary figures can be integrated in regular figure as well.

 According to Reviewer’s advice, our figures were reorganized in the new version of our MS.

Four previous supplementary figures are now integrated in regular figures in the present version of our MS.

It concerns:

- Figure #1 (previous figure S1): Early glycation product detection by using fluorescent boronic acids.

- Figure 2C and 2D (previous figure S3): Enhanced AGE formation in glycated erythrocytes.

- Figure 5E (previous figure S5): Enhanced oxidative stress in erythrocytes from diabetics.

- Figure 5G (previous figure S6): Enhanced AGE content in erythrocytes from diabetics.

[1]	Friguet, B. Oxidized protein degradation and repair in ageing and oxidative stress. *FEBS Lett* **580:**2910-2916; 2006.
